# Burnout, associated comorbidities and coping strategies in French community pharmacies—BOP study: A nationwide cross-sectional study

**DOI:** 10.1371/journal.pone.0182956

**Published:** 2017-08-11

**Authors:** David Balayssac, Bruno Pereira, Julie Virot, Aurore Collin, David Alapini, Damien Cuny, Jean-Marc Gagnaire, Nicolas Authier, Brigitte Vennat

**Affiliations:** 1 Université Clermont Auvergne, Inserm U1107, NEURO-DOL, UFR de Pharmacie, CHU Clermont-Ferrand, Clermont-Ferrand, France; 2 CHU Clermont-Ferrand, Clermont-Ferrand, France; 3 Université Clermont Auvergne, UFR de Pharmacie, Clermont-Ferrand, France; 4 Université Clermont Auvergne, Inserm U1107, NEURO-DOL, UFR de Pharmacie, Clermont-Ferrand, France; 5 Ordre des Pharmaciens—Conseil Régional Nord Pas de Calais, Lille, France; 6 Univ. Lille, CHU Lille, Institut Pasteur de Lille, EA 4483—IMPECS—IMPact de l’Environnement Chimique sur la Santé humaine, Lille, France; 7 Ordre des Pharmaciens—Conseil Régional Auvergne, Clermont-Ferrand, France; 8 Université Clermont Auvergne, Inserm U1107, NEURO-DOL, UFR de Médecine, CHU Clermont-Ferrand, Clermont-Ferrand, France; 9 Université Clermont Auvergne, UFR de Pharmacie, unité ACCePPT, Clermont-Ferrand, France; Universita degli Studi di Parma, ITALY

## Abstract

**Background:**

Work-related stress and burnout syndromes are unfortunately common comorbidities found in health professionals. However, burnout syndrome has only been partly and episodically assessed for community pharmacists whereas these professionals are exposed to patients’ demands and difficulties every day. Prevalence of burnout, associated comorbidities and coping strategies were assessed in pharmacy teams (pharmacists and pharmacy technicians) in French community pharmacies.

**Methods:**

This online survey was performed by emails sent to all French community pharmacies over 3 months. The survey assessed the prevalence of burnout (Maslach Burnout Inventory—MBI—questionnaire), anxiety, depression and strategies for coping with work-related stress.

**Results:**

Of the 1,339 questionnaires received, 1,322 were completed and useable for the analysis. Burnout syndrome was detected in 56.2% of respondents and 10.5% of them presented severe burnout syndrome. Severe burnout syndrome was significantly associated with men, large urban areas and the number of hours worked. Depression and anxiety were found in 15.7% and 42.4% of respondents, respectively. These co-morbidities were significantly associated with severe burnout syndrome. Higher MBI scores were significantly associated with medical consultations and medicinal drug use. Conversely, respondents suffering from burnout syndrome declared they resorted less to non-medical strategies to manage their work-related stress (leisure, psychotherapy, holidays and time off).

**Conclusion:**

This study demonstrated that community pharmacists and pharmacy technicians presented high prevalence of burnout syndrome, such as many healthcare professionals. Unfortunately, burnout syndrome was associated with several comorbidities (anxiety, depression and alcohol abuse) and the consumption of health resources. The psychological suffering of these healthcare professionals underlines the necessity to deploy a strategy to detect and manage burnout in community pharmacy.

## Introduction

Work-related stress and burnout syndromes are unfortunately common comorbidities found in health professionals [1–3]. Burnout syndrome is mainly defined and assessed through three dimensions including emotional exhaustion, depersonalization (i.e. cynicism) and the reduction of personal accomplishment that develop in response to chronic work-related stress [4]. The Maslach Burnout Inventory (MBI) [5] is the self-administered questionnaire used most in the scientific literature [6–8]. Burnout syndrome has a negative impact on jobs, decreasing motivation and performance [9]. In addition, burnout syndrome may also be associated with several disorders and negative consequences such as depression [6], anxiety [10], sleep disorders [11,12], fatigue [11], cardiovascular diseases [13,14], work-home conflict [15], substance abuse [16] and suicidal ideation [17]. Among common mental health problems associated to work, anxiety and depression are the main prevalent pathologies and moderate level evidence has been identified with workplace [18]. Burnout syndrome is also associated to alcohol dependence [19] and to increased uses of tobacco, alcohol and psychotropic drugs [1].

Burnout syndrome in health professionals has been widely studied, for example, a study revealed that more than half of US physicians met the criteria for burnout syndrome [20]. More worrying is that the prevalence of burnout for these US physicians increased by 10% between 2011 and 2014 [20]. Burnout syndrome in physicians can be attributed to several causatives factors such as excess workload, inefficiency, loss of autonomy, lack of meaning in work, increase of work productivity, malpractice, patients’ distress, and an unaccomplished personal life [21].

Workplace stress can be controlled by coping strategies, such as proposed by the American Psychological Association: track your stressors (identifying workplace stress situation…), develop healthy responses (good-quality sleep, exercise…), establish boundaries between work and home life (not to check email from home…), take time to recharge (time off…), learn how to relax (meditation, deep breathing exercises…), talk to your supervisor, get some support [22].

Community pharmacists are health professionals with the same work pressure as physicians such as being effective and avoiding mistakes in the everyday practice. In France and in several countries, community pharmacists are entrepreneurs who have engaged more than 1 million euros for a community pharmacy, and worried about the viability of their business. The community pharmacy world is changing in UK and in France, where the activities get harder, with budget cuts and regulatory changes [23,24]. Also, in a recent English study, community pharmacists had significantly higher levels of workplace stress than other health professionals [25]. Pharmacists are the guardians of medication safety, but this work-related stress in community pharmacies impacts the safety culture assessments and learning from quality related events. Moreover, pharmacists feel that efforts made were greater than the rewards realized, with implications for high stress [26]. Some specific sources of stress have been identified such as staff (competence shortage, confidence loss), interruptions (disruption of work flow), lack of breaks (impossibilities to break away from work), pharmacy environment (lack of privacy both for pharmacists and patients), isolation (lack of contact with other pharmacists), patient/public (very demanding and impatient) and difficulties to find the time to complete continuing professional development [27]. Beside work-related stress, burnout syndrome has only been partly and episodically assessed for community pharmacists whereas these professionals are exposed to patients’ demands and difficulties every day. To our knowledge, only one study has assessed burnout syndrome in a small number of Turkish community pharmacists (N = 251) [28]. In this study, community pharmacists had a very low level of personal accomplishment (71.3%) assessed through the MBI [28].

The main objective of this study was to assess the prevalence of burnout syndrome in French community pharmacies using a nationwide cross-sectional study. Secondary objectives were to assess comorbidities (anxiety and depression) and coping strategies.

## Methods

### Study design

We conducted a cross-sectional nationwide online survey of community pharmacies to assess burnout in pharmacy teams from April 17, 2015 to July 17, 2015 (3 months). The address of the online survey (https://redcap.chu-clermontferrand.fr/surveys/?s=B6W6pZfp8T) was sent by email via the National Order of Pharmacists to all French community pharmacies. In 2016, 22,104 community pharmacies have been counted in France (data from the National and the Regional Orders of Pharmacists). Individuals were included in the survey if they were pharmacists (owner or assistant) or pharmacy technicians working in a community pharmacy at the time of the survey. These professionals were defined as the pharmacy team and were asked to answer the online survey. Exclusion criteria were defined as follows: pharmacy students or other professionals (e.g., dietician, beautician) and lack of professional status information. There was no age limitation.

The study was made to conform to the STROBE (Strengthening the Reporting of Observational Studies in Epidemiology) guidelines for reporting observational studies [29]. The study was registered with the local correspondent of the French Commission on Information Technology, Data Files and Civil Liberty (Commission Nationale de l’Informatique et des libertés, n°0113) in accordance with French law and declared to the local ethics committee (Comité de Protection des Personnes sud-est 6, IRB: 00008526). Consent was obtained with the answer to the questionnaires.

### Survey protocol

The survey was anonymous and designed to record the following items: socio-demographic factors (age, sex, marital status, tobacco and alcohol habits); professional status (pharmacist or pharmacy technician, type of employment contract, number of hours worked per week); community pharmacy (number of customers/patients per day in the community pharmacy, number of citizens in the area of the community pharmacy); burnout syndrome using the Maslach Burnout Inventory (MBI) [5]; anxiety and depression symptoms using the hospital anxiety and depression scale (HADS) [30]; and strategies to manage work-related stress. These strategies were proposed to responders as medical consultation medication use (homeopathy, phytotherapy, anxiolytic and hypnotic medications), non-medical strategies (nutritional, relaxation and psychotherapy strategies), leisure, sport activities and holidays or time off. The duration of the online answer was estimated at 20 min.

Study data were collected and managed using REDCap electronic data capture tools hosted at CHU Clermont-Ferrand [31]. REDCap (Research Electronic Data Capture) is a secure, web-based application designed to support data capture for research studies, providing: 1) an intuitive interface for validated data entry; 2) audit trails for tracking data manipulation and export procedures; 3) automated export procedures for seamless data downloads to common statistical packages; and 4) procedures for importing data from external sources.

The principle objective was assessed using a validated questionnaire (MBI) [5]. Nonetheless, the differences between burnout syndrome and work-related stress are very narrow for most people (even health professionals) and we are convinced that people do not distinguish between either syndromes. Thus, in order to simplify the survey and avoid misinterpretations, we chose to talk about work-related stress instead of burnout when assessing coping strategies. Any worker can experience work-related stress without systematically being subject to burnout syndrome, whereas workers suffering from burnout syndrome experience work-related stress [32].

### Burnout, anxiety and depression questionnaires

The MBI has been designed to assess burnout syndrome in health professionals [5]. A French version of the MBI was used as described by Mion et al. [33]. The MBI is a 22-item questionnaire divided into 3 dimensions corresponding to emotional exhaustion (EE), depersonalization (DP) and personal accomplishment (PA). Each item is associated with a 7-point Likert scale. The following thresholds were used for EE (high ≥30, moderate 18–29 and low ≤17), DP (high ≥12, moderate 6–11 and low ≤5) and PA (high ≥40, moderate 34–39 and low ≤33). Burnout was defined for at least 1 dimension with a high score for EE and DP and a low score for PA). Burnout severity was associated with the number of dimensions with a high score (low score of PA) (low: 1/3 dimension, moderate: 2/3 dimensions and severe: 3/3 dimensions) [5].

The HADS was designed to assess anxiety and depressive disorders in patients. The HADS is a 14-item questionnaire divided into 2 subscales: 7 items for anxiety and 7 items for depression. For each subscale, a total score of ≤7 is considered normal, 8–10 is borderline or suggestive of possible anxiety/depression, and ≥11 is indicative of mood disorder or pathology [30].

### Statistical analysis

Statistical analysis was performed using Stata 13 (StataCorp, College Station, TX). The tests were two-sided, with a type-I error set at α = 0.05. Quantitative data were expressed as mean and standard deviation or median and interquartile range according to statistical distribution. The assumption of normality was studied using the Shapiro-Wilk test. Quantitative data were compared between independent groups using ANOVA or the Kruskal-Wallis test when the ANOVA assumptions were not satisfied, followed by the appropriate multiple comparison post-hoc test: Tuckey-Kramer or Dunn’s tests. The assumption of homoscedasticity was studied using the Bartlett test. Comparisons between groups concerning categorical data were performed using Chi-squared or Fisher’s exact tests followed when appropriate by Marascuillo’s procedure. In multivariate context, polynomial ordinal regression model was performed considering covariates retained according to univariate results and clinical relevance: gender, age and tobacco and alcohol consumption. As proposed by certain statisticians, we chose to report all the individual p-values without applying any mathematical correction for distinct tests comparing groups. Specific attention was given to the magnitude of improvement and to clinical relevance [34,35]. Finally, a sensitivity analysis has been applied to measure the impact of missing data on results, particularly for multivariate analysis, for which less than 10% of data were missing. Data were replaced using multiple imputation data approach.

## Results

### Sample description

Three months of data collection resulted in the collection of 1,339 answers and 1,322 answers pertaining to the definition of professional status of pharmacist (owner or assistant) or pharmacy technician were included for the analysis. In France, 27,390 owner-pharmacists and 27,327 assistant pharmacists have been counted in 2014 [36], representing a response rate of 3.1% (853) and 1.0% (288), respectively. No information is available on the number of pharmacy technicians in France. Descriptions of respondents and community pharmacies are presented in the Table 1. Owner-pharmacists were significantly older than assistant pharmacists and pharmacy technicians (F = 118.9, df = 1315, p<0.001). Women were significantly more represented in each professional category (owner-pharmacists: 58.9%, assistant pharmacists: 77.2% and pharmacy technicians: 91.7%, N = 1315) (Chi-squared = 89.1, df = 2, p<0.001).

**Table 1:** Description of the population of respondents in community pharmacies.

Professional status	Total	1,322 (100%)
	Pharmacists (owners)	853 (64.5%)
	Pharmacists (assistants)	288 (21.8%)
	Pharmacy technicians	181 (13.7%)
	Weekly working hours (median, IQR)	45, 35, 50
	Permanent employment contract	95.4% (1,255/1,316)
**Community pharmacy characteristics**	Customers/patients per day (median, IQR)	150, 100, 200
	Inhabitants in the area per 1000 inhabitants (median, IQR)	7, 2.6, 25
**Socio-demographic factors**	Age (years) (mean (sd))	45.1 (10.6)
	Gender (women)	67.4% (886/1,315)
	Living with a partner	81.5% (1061/1,302)
	Single	17.1% (223/1,302)
	Widower	1.4% (18/1,302)
	Parent (at least 1 child)	76.6% (1,006/1,313)
	Tobacco	14% (182/1,298)
	Alcohol consumption (occasionally)	72.9% (951/1,304)
	>3 alcohol units/day (male)	7.4% (26/351)
	>2 alcohol units/day (female)	3.4% (20/594)

For alcohol consumption, World Health Organization (WHO) limits were defined for males as >3 units/day or >21 units/week and for female: >2 units/day or >14 units/week.

### Burnout syndrome

Mean score of EE, DP and PA were 23.7±14.7, 8.8±7.3 and 37.1±8.1, respectively. Of the respondents, 36.7% (485/1,322) presented a high EE score, 30.8% (407/1,322) a high DP score and 27.2% (360/1,322) a low PA score. According to the MBI scoring, 43.8% of respondents (579/1,322) had no burnout. Mild burnout syndrome (at least 1 dimension with a high EE or DP score, or a low PA score) was identified in 28.2% of respondents (373/1,322), moderate burnout syndrome (2 dimensions with a high EE and/or DP score, and/or a low PA score) in 17.5% (231/1,322) and severe burnout syndrome (3 dimensions with a high EE and DP score, and a low PA score) in 10.5% (139/1,322). The following factors: age, professional status (owner-pharmacist, assistant pharmacist or pharmacy technician) and number of patients/customers per day had no impact on MBI scores. However, men were subject to significantly higher burnout severity than women (Chi-squared = 26.4, df = 3, p<0.001). Respondents working in a large urban area (i.e. large population) had higher burnout severity (Kruskal-Wallis test = 11.9, df = 3, p = 0.008). In respondents with burnout syndrome, severe burnout was related to the number of hours worked (Kruskal-Wallis test = 11.2, df = 3, p = 0.01).

### Comorbidities

**Fig 1:**
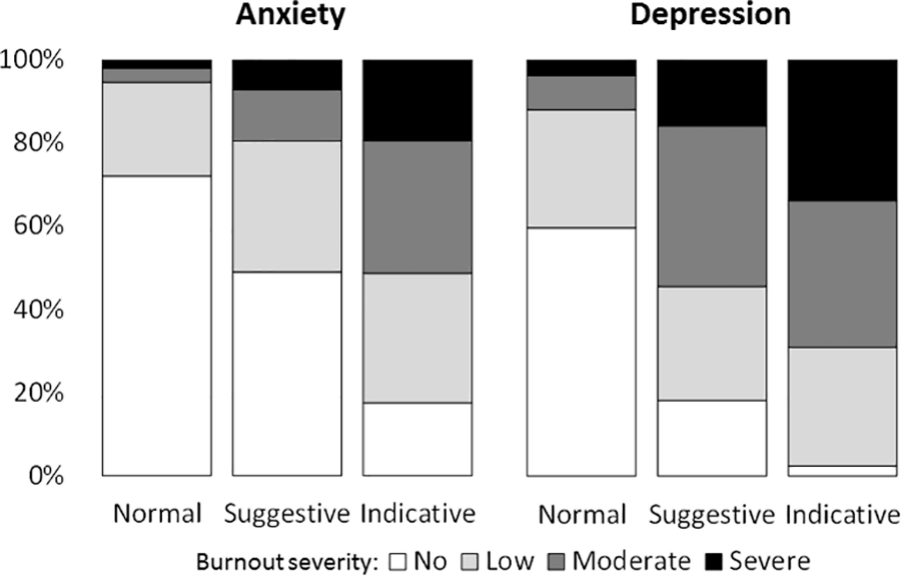
Distribution of burnout severity between anxiety and depression screening (HADS). Burnout syndrome severity was described as no burnout, low, moderate or severe according to the MBI questionnaire. Screening of anxiety and depression was assessed using the HADS questionnaire and described as normal, suggestive or indicative. Burnout severity was significantly associated with anxiety and depression scores (normal, suggestive and indicative) (p<0.001).

According to HADS scoring, 22.1% (292/1,322) and 42.4% (561/1,322) of respondents had suggestive and indicative scores of anxiety, and 16.6% (220/1,322) and 15.7% (207/1,322) had suggestive and indicative scores of depression, respectively. Higher HADS scores for anxiety or depression were significantly related to burnout severity (Chi-squared = 387.1, df = 6, p<0.001 for anxiety and Chi-squared = 455.2, df = 6, p<0.001 for depression) (Table 2 and Fig 1).

**Table 2:** Mean scores (standard deviation) of anxiety (HADS) and depression (HADS) levels related to burnout severity.

Items	Total	Burnout severity				p-value
		No	Low	Moderate	Severe	
**Anxiety**	9.79 (4.79)	7.00 (3.49)	10.39 (4.58)	13.36 (3.86)	13.81 (3.95)	p<0.001^a^^,^^b^^,^^c^^,^^d^^,^^e^
**Depression**	5.72 (4.31)	3.04 (2.61)	6.02 (3.86)	9.04 (3.56)	10.54 (3.89)	p<0.001^a^^,^^b^^,^^c^^,^^d^^,^^e^^,^ p<0.01^f^

a: No burnout *vs* low burnout

b: No burnout *vs* moderate burnout

c: No burnout *vs* severe burnout

d: low burnout *vs* moderate burnout

e: low burnout *vs* severe burnout

f: moderate burnout *vs* severe burnout

**Fig 2:**
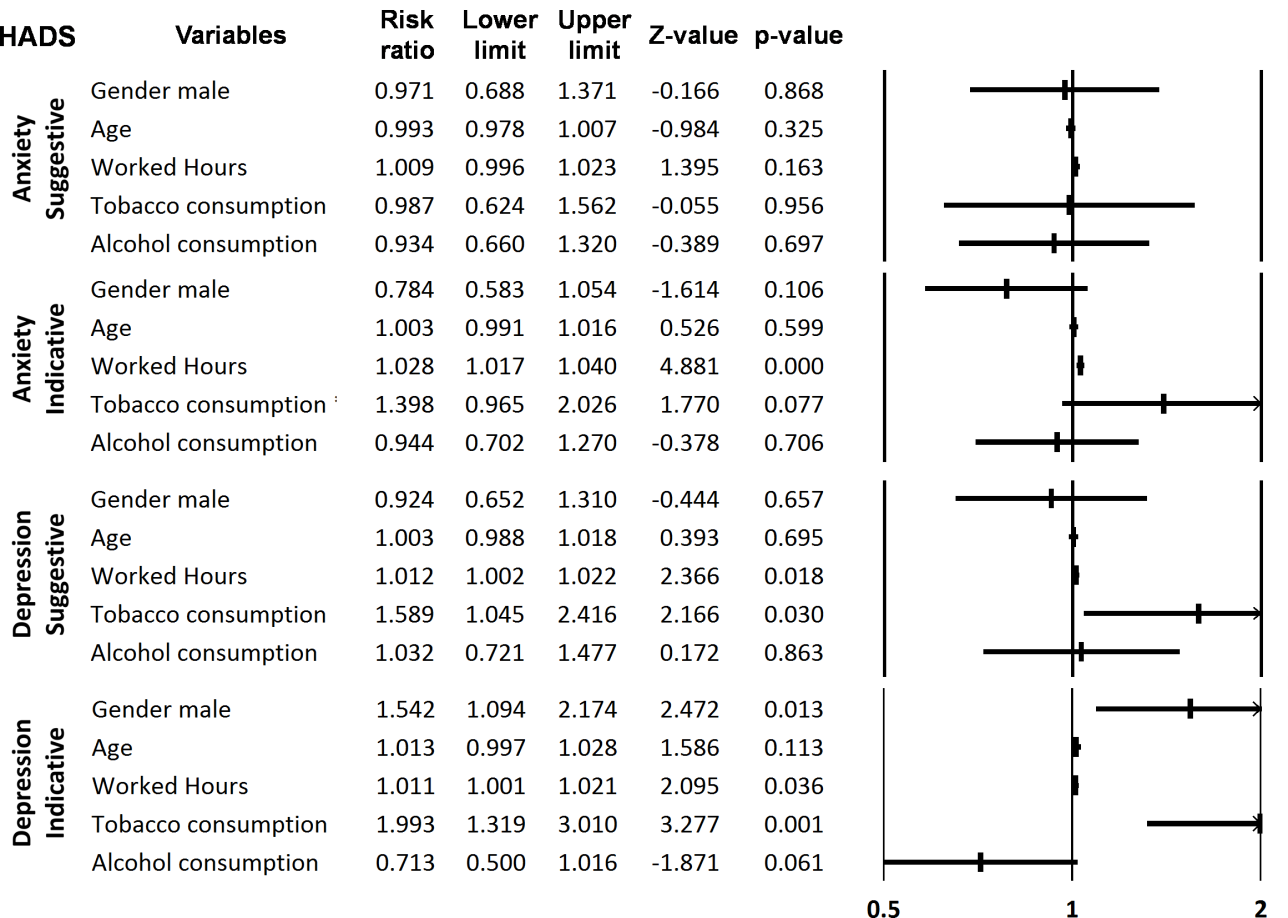
Forrest plot of the multivariate analysis of the relation between anxiety or depression (suggestive and indicative scores, HADS) and comorbidities (tobacco and alcohol consumptions), worked hours, age and gender (Risk ratio and confident interval 95%).

HADS scores for depression were significantly higher for men than women (Chi-squared = 10.0, df = 2, p = 0.007), but there was no difference between gender for the anxiety scores. HADS scores for anxiety and depression were related to the number of hours worked per day (Kruskal-Wallis test = 29.2, df = 2, p<0.001 for anxiety and Kruskal-Wallis test = 25.6, df = 2, p<0.001 for depression). HADS scores for anxiety and depression were also related to alcohol consumption above the WHO limits in men (Chi-squared = 12.6, df = 2, p = 0.002 and Chi-squared = 11.1, df = 2, p = 0.004, respectively), but not in women. HADS scores of anxiety and depression were not related to respondents’ age, job status, the number of customers/patients per day or the number of inhabitants in the community pharmacy area. Using a multivariate analysis, HADS scores were compared to gender, age, number of hours worked, tobacco and alcohol consumptions. Number of hours worked was significantly related to anxiety (indicative scores) and depression (suggestive and indicative scores). Males were significantly associated to indicative score of depression. Tobacco consumption was related to suggestive and indicative scores of depression (Fig 2).

The frequency and quantity of daily tobacco smoking were not related to burnout syndrome (Table 3). Alcohol consumption was not related to burnout syndrome, but alcohol consumption above the World Health Organization (WHO) limits for males was associated with burnout severity (Chi-squared = 14.1, df = 3, p = 0.003) (Table 3). Finally and more worryingly, we observed that 8.2% of respondents (106/1,300) declared that they used psychoactive drugs (alcohol or illicit drugs) to manage their work-related stress, particularly for the higher MBI scores (Chi-squared = 48.5, df = 3, p<0.001) (Table 3).

**Table 3:** Tobacco, alcohol and psychoactive drugs consumption related to burnout severity.

	Total	Burnout severity				p-value
		No	Low	Moderate	Severe	
Tobacco smoking	14.0% (182/1,298)	13.0%	13.6%	15.8%	16.2%	-
<1 cigarette pack/day	84.9% (152/179)	83.3%	88.0%	86.1%	81.0%	-
1–2 cigarette packs/day	15.1% (27/179)	16.7%	12.0%	13.9%	19.0%	
Alcohol consumption	72.9% (951/1,304)	73.9%	71.4%	74.3%	70.5%	-
>3 alcohol units/day (male)	8.8% (26/295)	1.9%	7.9%	17.2%	15.8%	p<0.05^a^
>2 alcohol units/day (female)	3.9% (20/510)	2.7%	7.3%	1.2%	7.1%	-
Psychoactive drugs	24.5% (318/1,300)	4.0%	7.1%	12.3%	20.9%	p<0.01^a^^,^^c^, p<0.001^b^

Percentages are expressed for each class of burnout severity (no burnout, low, moderate and severe, respectively). For alcohol consumption, World Health Organization (WHO) limits were defined for males as >3 units/day or >21 units/week and for female: >2 units/day or >14 units/week.

a: No burnout *vs* moderate burnout

b: No burnout *vs* severe burnout

c: low burnout *vs* severe burnout

**Fig 3:**
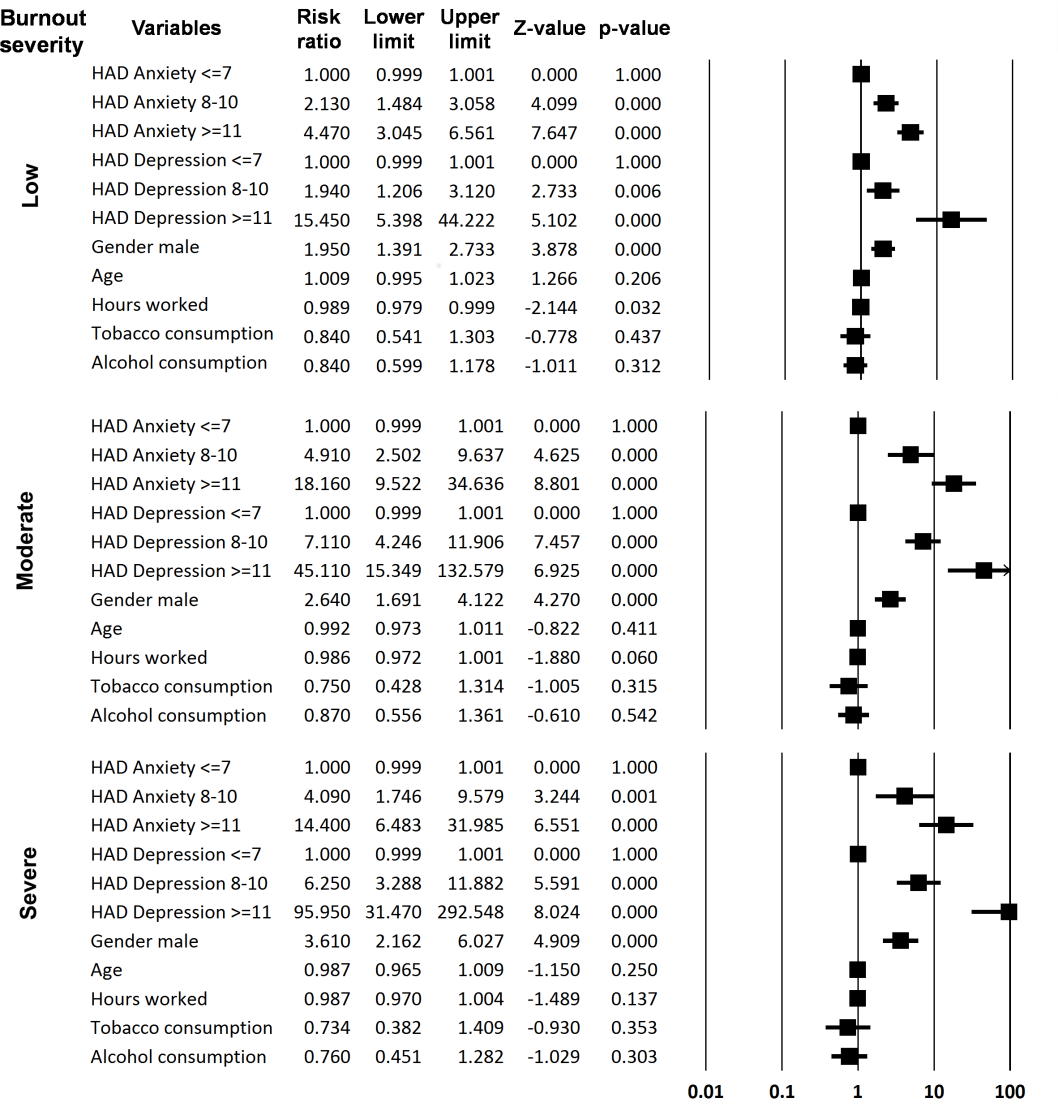
Forrest plot of the multivariate analysis of the relation between burnout syndrome severity and comorbidities (HAD scale for anxiety and depression, tobacco and alcohol consumptions), worked hours, age and gender (Risk ratio and confident interval 95%).

Using a multivariate analysis, burnout syndrome severity was compared to comorbidities (anxiety and depression), age and gender. The ages of the respondents were not related to burnout severity, as described with the univariate analysis. However, men have a higher risk of developing burnout syndrome in both the univariate and multivariate analyses. All the grades of burnout severity were related to depressive disorders (indicative or suggestive scores of the HADS), whereas only the highest scores (indicative) for anxiety were related to all the grades of burnout severity. Suggestive scores of HADS for anxiety were related to low and moderate burnout (Fig 3).

### Strategies to improve work-related stress

Medical and non-medical strategies to improve work-related stress are presented in Table 4. Respondents with higher MBI scores (all, men or women) resorted significantly more to medical consultations to manage their work-related stress than respondents with lower MBI scores (Chi-squared = 50.4, df = 3, p<0.001). Interestingly, 68.0% of men (17/25) with moderate to severe burnout resorted to medical consultations while this figure was 46.2% of women (48/104). A quarter of respondents took medications (homeopathy, phytotherapy, anxiolytic or hypnotic drugs) to improve their work-related stress. Anxiolytic and hypnotic drugs represented 75% of the medications used. Higher MBI scores were related to medication use (Chi-squared = 94.7, df = 3, p<0.001). Of the respondents who take either anxiolytic or hypnotic drugs to manage their work-related stress, 52.3% (79/151) and 59.1% (39/66) are self-medicated, respectively. Even if burnout severity was significantly different among respondents using self-medication or medical prescription (Chi-squared = 8.1, df = 3, p = 0.04), no clear relation was observed between high MBI scores and self-medication.

**Table 4:** Medical and non-medical management of workplace stress related to burnout severity.

	Total	Burnout severity				p-value
		No	Low	Moderate	Severe	
Medical consultation	9.8% (129/1,315)	3.8%	11.4%	16.5%	19.4%	p<0.001^a^^,^^b^^,^^c^
Medication use	24.1% (314/1,302)	12.2%	27.2%	37.7%	42.4%	p<0.001^a^^,^^b^^,^^c^, p<0.05^d^
Self-medication	64.4% (197/306)	73.9%	59.4%	69.8%	52.7%	-
Medical prescription	35.6% (109/306)	26.1%	40.6%	30.2%	47.3%	-
Homeopathy medication	2.6% (8/307)	5.9%	1.1%	3.5%	0.0%	-
Phytotherapy medication	25.4% (78/307)	48.5%	21.5%	19.5%	13.6%	p<0.01^a^^,^^b^, p<0.001^c^
Anxiolytic medication	50.2% (154/307)	38.2%	53.8%	54.0%	52.5%	-
Hypnotic medication	21.8% (67/307)	7.4%	23.7%	23.0%	33.9%	<0.01^a^^,^^b^^,^^c^
Leisure	72.8% (947/1,301)	82.8%	67.1%	67.0%	55.8%	p<0.001^a^^,^^b^^,^^c^
Sport activities	54.7% (705/1,289)	60.8%	54.0%	48.5%	41.9%	p<0.01^b^^,^^c^
Holidays or time off	43.3% (562/1,299)	49.0%	43.6%	36.0%	30.4%	p<0.01^b^, p<0.001^c^, p<0.05^d^
Nutritional strategies	24.5% (318/1,300)	24.2%	24.5%	24.6%	25.2%	-
Relaxation strategies	14.3% (186/1,304)	11.8%	16.4%	15.0%	17.5%	-
Psychotherapy	7.9% (103/1,301)	4.2%	11.1%	11.0%	10.1%	p<0.01^a^^,^^b^

Percentage of responders for self-medication, medical prescription and medication type (homeopathy, phytotherapy, anxiolytic, hypnotic) are expressed for medication users (N = 314). Percentages are expressed for each class of burnout severity (no burnout, low, moderate and severe, respectively).

a: No burnout *vs* low burnout

b: No burnout *vs* moderate burnout

c: low burnout *vs* severe burnout

d: low burnout *vs* severe burnout

Almost three quarters of respondents declared that they use non-medical strategies to manage their work-related stress (mainly leisure and sport activities) (Table 4). However, individuals suffering from burnout syndrome (low to severe) were less involved in non-medical strategies to manage their work-related stress, such as leisure activities (Chi-squared = 59.0, df = 3, p<0.001), sport activities (Chi-squared = 20.9, df = 3, p<0.001), psychotherapy (Chi-squared = 19.5, df = 3, p<0.001) and holidays or time off (Chi-squared = 21.8, df = 3, p<0.001). Resorting to nutritional and relaxation strategies was not related to burnout syndrome.

## Discussion

The BOP study is the first nationwide survey assessing burnout syndrome in community pharmacies. Of the pharmacy team (pharmacists and pharmacy technicians), 56.2% of individuals presented burnout syndrome (low to severe), and 10.5% of respondents had severe burnout syndrome (high score for EE and DP, a low score for PA). Severe burnout syndrome (high scores for EE and DP and a low score for PA) was identified for 12% of European family doctors [1] and 28.9% of Irish and UK urologists [37]. The high scores of EE and DP in the BOP study (36.7% and 30.8%, respectively) were close to data from the literature, such as for Irish urologists (28.6% and 26.9%; N = 575) [37], European family doctors (43% and 35.3%; N = 1393) [1], nurses from eight countries (35.1% and 31.3%; N = 750) [38] and health professionals from south and southeast Europe (31.9% and 33.2%; N = 2,623) [39]. However, low PA scores seemed to be less frequent in the BOP study (27.2%) compared to the literature 40.3% (N = 575) [37], 39.5% (N = 1,393) [1] and 28.4% (N = 750) [38]. In the only study assessing burnout syndrome in community pharmacies (Ankara, Turkey), by Calgan *et al*., pharmacists had a low level of burnout (1.2% with a high level of EE and 0.8% with a high level of DP), but more surprisingly 71.3% of them had a low level of PA. But in this study, no global score was described to assess burnout severity [28].

In French community pharmacy teams, burnout syndrome was significantly associated with anxiety and depression. More worryingly, these comorbidities remained globally high in this population, with approximately ≈33% of responders suffering from depression and ≈66% suffering from anxiety. Burnout syndrome and depression are very closely related [40]. Depression could be related to an exacerbation of burnout symptoms or to a clinical form of burnout [41]. However, the scientific literature remains equivocal on the temporal link between depression and burnout development [6,8,41]. It has been suggested that stress and anxiety are associated with the early stage of burnout [41]. There is also a strong overlap between depression and anxiety [42]. In this population of community pharmacists and pharmacy technicians, burnout syndrome was strongly associated with depression and anxiety, although it was not the aim of the present study to distinguish the occurrence of burnout and these comorbidities.

Sex difference is particularly surprising in this study. Men suffered significantly more of burnout syndrome, depression and alcohol abuse than women. No unequivocally explanation has been proposed. There were significantly more women than men in each professional category (owner- and assistant pharmacists, pharmacy technicians), but sex ratio was more equilibrated for owner-pharmacists (women: 58.9%). Consequently, it was suggested that these men were more exposed to entrepreneur’s burden. Data from the literature shown that both men and women are affected by work-related stress, but gender differences exist only for specific components or consequences of work related stress. Rivera-Torres et al. have reviewed these gender specificities. There is no difference between women and men for personal accomplishment, self-esteem or well-being. For burnout, gender differences are debated and unclear. Women seem to suffer more from mental disorders, depression, anxiety and psycho-somatic illnesses than men. Interestingly, men seem to experience higher levels of stress [43].

Patients suffering from burnout syndrome resorted more to medical consultations, medications and psychoactive drugs, which have already been described in previous publications [37,44]. In medication users, burnout syndrome had no impact on the rate of self-medication and medical prescription, whereas we could have expected that in this population of pharmacists with easy access to medication, self-medication would have been higher in patients suffering from burnout syndrome. However, half of the respondents took anxiolytic and hypnotic medication without medical prescription. The rate of psychoactive drug users (8.2%) remained similar and even slightly lower according to the data available on healthcare professionals (10–15%) [37,45]. Fortunately, among the proposed coping strategies, leisure, sport and time-off were the most prominent for all the respondents. It seems that work-related stress is a strong leitmotiv for these activities. A great number of studies have demonstrated the anxiolytic and anti-depressant effects of physical activity [46]. The positive effects of vacations have been demonstrated on health and well-being, but these effects are short lived after return to work [47]. Individuals suffering from burnout syndrome declared less recourse to these non-medical strategies. Burnout has already been negatively associated with frequent exercise [39]. Stults-Kolehmainen and Sinha have reviewed the effects of stress on physical activity and demonstrated that stress impedes individuals’ efforts to be more physically active [46]. To our point of view, this decrease in recourse to positive activities such as holidays, sport and leisure, could be the consequence of anhedonia associated with burnout syndrome, but this remains to be specifically demonstrated.

The study was performed over a short period of 3 months from mid-April to mid-July 2015, thus from the end of spring to the beginning of summer and a very clement period of the year in France, with an increase in temperature and longer days. Thus we can assume that this period may limit mood disturbance and burnout syndrome. Another limitation of the present study was the small sample size, which can’t reflect whole French community pharmacies. However, the sample studied mirrored the national population of pharmacists relatively closely, since women represented 68.1% of pharmacists in community pharmacies in France in 2014 [36] and 63.5% in our study. The mean age in France in 2014 was 49.9 years for owner-pharmacists and 43.5 years for assistant pharmacists, while in our study it was 48.2 (9.2) years and 39.3 (10.8) years, respectively. The online format of the survey may have favorited younger and “connected” persons. Respondents to the present survey were mainly owner-pharmacists (64.5%, assistant pharmacists 21.8% and pharmacy technicians 13.7%). This can be explained by the fact that the survey was sent by email to each community pharmacy, thus in most cases checked by the owner of the community pharmacy (owner-pharmacists). Therefore we can hypothesize that owner-pharmacists influenced the survey results with their specific problems as entrepreneurs, although there was no difference in burnout syndrome between professional statuses. Furthermore, the assessment of burnout syndrome with the MBI questionnaire may have introduced certain limitations mainly related to its scientific and even arbitrary construction, since burnout syndrome should be identified by a clinical diagnostic [8]. Finally, as many online survey, results might be biased by the responder himself who could have a strong interest in the topic, such as burnout syndrome.

Community pharmacists and pharmacy teams are exposed to burnout syndrome, as are other health professionals. This burnout syndrome is strongly associated with comorbidities (anxiety and depression) and health care consumption (medical consultations and medications). Strategies must be developed to help community pharmacy teams coping with burnout syndrome in order to counter comorbidities.

## Supporting information

S1 Table BOP database. (XLSX)
